# Domain-Specific Cognition Mediates the Association Between Mobility and Wayfinding Ability in Older Adults

**DOI:** 10.1093/geroni/igaf122.3748

**Published:** 2025-12-31

**Authors:** Alexis Chargo, Cheryl Dahle, Ana Daugherty

**Affiliations:** Wayne State University, Detroit, Michigan, United States; Wayne State University, Detroit, Michigan, United States; Wayne State University, Detroit, Michigan, United States

## Abstract

Older adults frequently report difficulties with community wayfinding, which may be further exacerbated by age-related mobility decline; however, the ways in which these processes intersect to support navigation as a fundamental behavior for independent functioning is poorly understood. The connection between mobility and wayfinding may be attributable to a shared set of cognitive processes that are vulnerable to decline. Thus, we examined the contributions of domain-specific cognition as a potential mediator between mobility and wayfinding in a community-based sample of older adults (N = 74, age 53-89 years). Participants underwent a comprehensive assessment of cognitive and mobility function and completed a seated virtual assessment of wayfinding with subsequent recall of environment details. A structural equation model was specified in which a latent mobility construct (indicators: balance, timed forward and backward walking) predicted recall of environment details via three cognitive mediators: processing speed (SDMT), visuospatial working memory (BVMT-R), and declarative memory (VAL-delay). The hypothesized model demonstrated excellent fit and accounted for 16.3% of the variance in environment recall (p = .04). While the total effect of mobility on recall of environment details was not significant (p = .072), evidence for mediation was supported by a significant cumulative indirect effect (p = .038). A comparison of the percentages of unique effects revealed processing speed as the largest contributor, followed by declarative and visuospatial working memory function. The results suggest a role of mobility in the acquisition and shaping of our memory for the environment that is explained in part by a shared cognitive-mobility architecture.

